# Predictive models for live birth outcomes following fresh embryo transfer in assisted reproductive technologies using machine learning

**DOI:** 10.1186/s12967-025-07045-6

**Published:** 2025-09-24

**Authors:** Shengnan Wu, Xinbo Wang, Yuechen Liu, Yongyong Ren, Mei Zhao, Haitao Song, Hao Shen, Yueting Wu, Zhiyun Wei, Hui Lu, Kunming Li

**Affiliations:** 1 Department of Integrated Traditional Chinese Medicine (TCM) & Western Medicine, Shanghai Key Laboratory of Maternal Fetal Medicine, Shanghai Institute of Maternal-Fetal Medicine and Gynecologic Oncology, Clinical and Translational Research Center, Shanghai First Maternity and Infant Hospital, School of Medicine, Tongji University, Shanghai, 201204 China; 2 Department of Bioinformatics and Biostatistics, School of Life Sciences and Biotechnology, Shanghai Jiao Tong University, Shanghai, 200240 China; 3 SJTU-Yale Joint Center for Biostatistics and Data Science, Technical Center for Digital Medicine, National Center for Translational Medicine, Shanghai Jiao Tong University, Shanghai, 200240 China; 4 Institute of Bioinformatics, Shanghai Academy of Experimental Medicine, Shanghai, 200240 China; 5 Shanghai Children’s Hospital, School of Medicine, Shanghai Jiao Tong University, Shanghai, 200062 China; 6 Center for Reproductive Medicine, Shanghai First Maternity and Infant Hospital, School of Medicine, Tongji University, Shanghai, 201204 China; 7 Shanghai Artificial Intelligence Research Institute, Shanghai, 200240 China; 8 XiangFu Laboratory, Jiashan, 314102 China; 9 Shanghai Key Lab of Reproduction and Development, Shanghai Key Lab of Female Reproductive Endocrine Related Diseases, Obstetrics & Gynecology Hospital of Fudan University, Shanghai, 200433 China; 10 Department of Reproductive Medicine, Shanghai Tenth People’s Hospital, Tongji University School of Medicine, Shanghai, 200072 China

**Keywords:** Assisted reproductive techniques, Ensemble learning, Prediction model, Pregnancy outcomes

## Abstract

**Background:**

Infertility affects approximately 15% of couples globally, with assisted reproductive technologies (ARTs) becoming the primary interventions. Despite the growing use of ARTs, success rates have plateaued at around 30%, highlighting the need for improved predictive models to enhance outcomes. This study aimed to develop a machine learning-based predictive model for live birth outcomes following fresh embryo transfer.

**Methods:**

A total of 51,047 ART records were collected from 2016 to 2023 at the Shanghai First Maternity and Infant Hospital. After data preprocessing, 11,728 records and 55 pre-pregnancy features were analyzed. Six machine learning models—Random Forest (RF), eXtreme Gradient Boosting (XGBoost), Gradient Boosting Machines (GBM), Adaptive Boosting (AdaBoost), Light Gradient Boosting Machine (LightGBM), and Artificial Neural Network (ANN)—were employed to construct the prediction model.

**Results:**

Among the models, RF demonstrated the best predictive performance, achieving an area under the curve (AUC) value exceeding 0.8. Key predictive features included female age, grades of transferred embryos, number of usable embryos, and endometrial thickness. A web tool was developed to assist clinicians in predicting outcomes and individualizing treatments based on patient data.

**Conclusions:**

This study presents a significant advancement in predicting live birth outcomes prior to embryo transfer, moving beyond traditional assessments. The findings underscore the potential of machine learning to improve clinical decision-making and enhance patient counseling in ARTs.

**Supplementary Information:**

The online version contains supplementary material available at 10.1186/s12967-025-07045-6.

## Background

Infertility is a crucial global health issue, affecting approximately 15% of couples worldwide [1]. Assisted reproductive techniques (ARTs) have emerged as the leading therapeutic interventions for addressing this condition [2]. The increasing number of reported ART treatments, particularly in vitro fertilization (IVF) and intracytoplasmic sperm injection (ICSI), has significantly contributed to the rise in birth rates over the past two decades [3]. However, the success rate of ARTs has plateaued at around 30% in recent years [4, 5], presenting a considerable challenge for couples facing infertility.

Enhancing the success rate of ARTs remains a critical objective for healthcare professionals specializing in reproductive medicine [6]. Recurrent implantation failure and miscarriage exert profoundly detrimental effects on patients. Therefore, the ability to predict ART outcomes in advance can assist clinicians in optimizing and adjusting treatment strategies, thereby reducing patients’ psychological distress and alleviating their financial burden. Traditionally, predictions regarding ART outcomes have relied on clinicians’ subjective assessments, primarily based on patient age and historical success rates at fertility centers [7]. Previous research has examined various factors influencing ART outcomes, including body mass index (BMI), anti-Müllerian hormone (AMH) levels [8–10], and the number of embryos transferred. Recently, artificial intelligence (AI) has emerged as a promising tool to enhance predictive accuracy by analyzing large datasets and identifying patterns overlooked by conventional methods [11]. Machine learning, a subfield of AI, enables systems to learn from data without explicit programming [12]. Its implementation facilitates the analysis of extensive medical data while reducing subjective biases [13], thereby refining predictive models for ART success [7, 14].

Fresh embryo transfer is the most basic initial treatment for ART, and it is also the first choice for young patients with good ovarian response. It requires rapid decision-making within 2–3 days after oocyte retrieval, thus there is an urgent need for timely prognostic tools to assist the transplantation strategy. Fresh embryo transfer can also shorten the treatment cycle and reduce the economic cost of treatment. Therefore, enhancing the live birth rate of fresh embryo transfer is critically important and holds substantial clinical significance. The successful delivery of at least one newborn depends on the effective establishment of pregnancy, which is influenced by numerous factors throughout the nearly ten-month gestation period. This complexity makes the prediction of successful delivery a challenging task. The objective of this study is to apply machine learning methods to develop a live birth prediction model for fresh embryo transfer, utilizing a comprehensive set of indicators collected during the ART procedure.

A total of 51,047 records were collected from 2016 to 2023 at the Shanghai First Maternity and Infant Hospital. After data preprocessing, 11,728 records with 75 pre-pregnancy features were included in the analysis. Six machine learning models—Random Forest (RF), eXtreme Gradient Boosting (XGBoost), Gradient Boosting Machines (GBM), Adaptive Boosting (AdaBoost), Light Gradient Boosting Machine (LightGBM), and Artificial Neural Network (ANN)—were employed to develop a clinical pregnancy outcome prediction model. To create a more concise model, 55 features were selected for further analysis. Among these models, RF demonstrated the best predictive performance, with an AUC exceeding 0.8, followed by XGBoost. Through a mechanistic analysis of the optimal model, RF, several features were identified as significant for both prediction and clinical practice, including female age, grades of transferred embryos, count of usable embryos, and endometrial thickness. Furthermore, the global impact of variations in these features on live birth outcomes was elucidated. In addition, a web tool was developed to assist physicians in predicting clinical outcomes and guiding individualized treatment plans. This study highlights the predictive value of pre-transplantation assessments, representing a substantial advancement over evaluations conducted during pregnancy, and offers valuable support for clinicians in providing personalized counseling prior to embryo transfer.

## Methods

### Data source and study design

Data were collected from patients undergoing assisted reproduction at Shanghai First Maternity and Infant Hospital between 2016 and 2023. This study focused exclusively on patients who underwent fresh embryo transfer. Clinical information and baseline characteristics were retrieved from the hospital’s internal database. The primary outcome of interest was live birth following pregnancy.

### Machine learning models

In this analysis, we evaluated six machine learning models for constructing classification predictive models: RF, XGBoost, GBM, AdaBoost, LightGBM, and ANN. Each model offers unique strengths and weaknesses that determine its suitability for specific tasks. RF is known for its robustness and interpretability, effectively handling diverse data types, although it can become complex and computationally intensive with large datasets. XGBoost achieves high predictive accuracy and incorporates regularization techniques to mitigate overfitting, yet it requires careful hyperparameter tuning and can be time-consuming to train. GBM is similarly effective in achieving high accuracy but is susceptible to overfitting without proper adjustments and typically requires longer training time. AdaBoost is straightforward to implement and focuses on misclassified instances, but it may struggle with noisy data and outliers. LightGBM offers significant efficiency and lower memory usage, making it ideal for large datasets, though it may sacrifice interpretability and requires precise parameter tuning. Finally, ANN is highly flexible and capable of modeling complex relationships, but it demands substantial computational resources and is prone to overfitting if not properly managed.

For these methods, we adopted a grid search approach to optimize hyperparameters using 5-fold cross-validation. The specific key hyperparameters are detailed in the Supplementary Methods. Specifically, the training data were split into five subsets. For each subset, the model was trained on the other four and tested on the remaining one, using the area under the receiver operating characteristic curve (AUC) as the evaluation metric. The AUC scores were averaged across all folds, and the hyperparameters yielding the highest average AUC were selected. The model was then retrained on the full training dataset. To evaluate performance, we plotted the ROC curve and calculated metrics like AUC, accuracy, kappa, sensitivity, specificity, precision, recall, and F1 score on the testing data. Furthermore, we performed sensitivity analysis, including subgroup analysis (stratified by key clinical variables) and perturbation analysis, to assess the stability and generalizability of the top-performing model.

### Model interpretation

To gain deeper insights into the underlying mechanisms of the prediction model, we identified the most influential features from the best-performing, more parsimonious model. This analysis revealed several key factors that contributed to the success rates of ART. Additionally, we provided a comprehensive explanation of the model’s mechanisms at both the dataset and instance levels. Specifically, for critical features, we examined the partial dependence (PD), local dependence (LD), accumulated local (AL) profile, and breakdown profile. The PD profile, widely used to visualize the marginal effects of predictors in black-box supervised learning models, may yield potentially misleading results due to unreliable extrapolation when predictors are highly correlated [15]. In contrast, the AL profile mitigates this issue by summarizing variable impact without requiring unreliable extrapolation in cases of correlated predictors. A comparative summary of the three interpretability techniques (PD, LD, and AL) is provided in the ​​Supplementary Table S2​​. Furthermore, we constructed 2D partial dependence plots to explore the interaction effects among several important features.

### Statistical analysis and machine learning platform

The primary analysis was conducted using R version 4.4. Machine learning algorithms, including random forest, GBM, and AdaBoost, were implemented primarily through the caret package (version 6.0-94). XGBoost was executed using the xgboost package (version 1.7.7), while LightGBM was implemented via the bonsai package (version 0.3.0). The ANN was developed in Python version 3.8, supported by Torch version 1.9.1.

### Ethical approval and compliance

The study protocol received ethical approval from the Ethics Committee of Shanghai First Maternity and Infant Hospital (Approval Number: KS22368). All data were rigorously anonymized prior to analysis to ensure complete protection of patient confidentiality.

## Results

### Data inclusion criteria and filtering

**Fig. 1:**
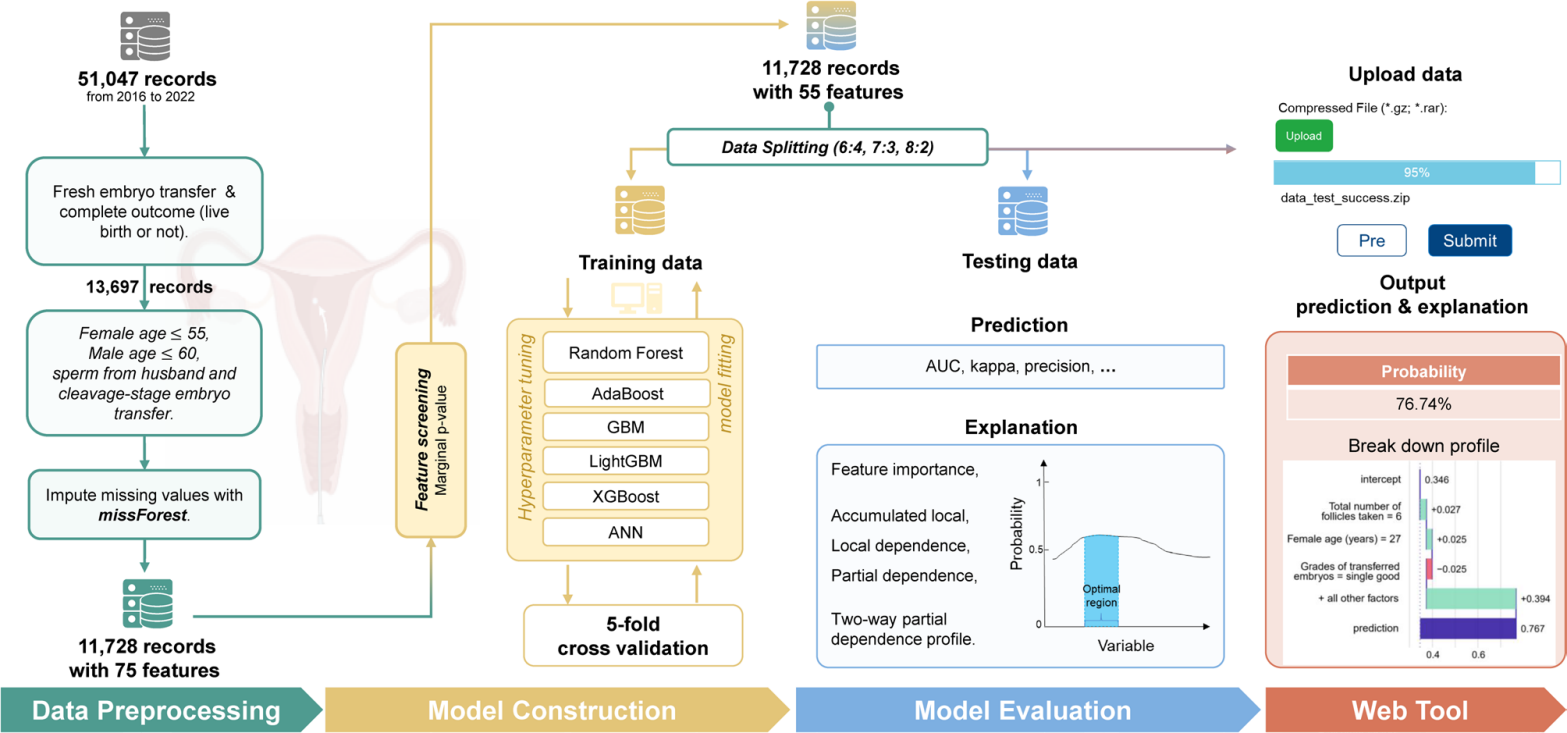
Workflow of study design

The original dataset consisted of 51,047 records. To refine the analysis, we focused on patients who used fresh embryos and had fully tracked outcomes, resulting in 13,362 records. Additionally, we keep records with female age $\:\le\:$ 55, male age $\:\le\:$ 60 years, sperm from the husband and cleavage-stage embryo transfer, yielding a final dataset of 11,728 records (Fig. 1). A total of 75 features were retrieved. Missing values were imputed using the nonparametric method *missForest* [16], which is efficient particularly in the case of mixed-type data.

### Basic characteristics of the dataset

The records were classified into two groups: live birth (*n* = 3,971; 33.86%) or not (*n* = 7,757; 66.14%). For each group, categorical variables were summarized as frequencies (percentages), while continuous variables were reported as means (standard deviations). Intergroup differences were assessed using the chi-square test for categorical variables and the non-parametric Kruskal-Wallis test for continuous variables. To balance predictive accuracy with model parsimony, we developed a tiered feature selection protocol: after establishing a baseline model with all 75 predictors (Supplementary Table S1), features were retained through ​​(i)​​ data-driven criteria (p $\:\le\:$ 0.05 or top-20 Random Forest importance ranking) followed by ​​(ii)​​ clinical expert validation to eliminate biologically irrelevant variables and reinstate clinically critical features. This yielded a final model with 55 clinically and statistically validated predictors, detailed in Table 1.

**Table 1:** Characteristics of the refined feature set (55 features). Categorical variables are summarized as frequency (percentage), with intergroup differences assessed using the chi-square test. Continuous variables are reported as mean (standard error), and differences between groups are evaluated using the Kruskal-Wallis test. Features are ranked by ascending p-values, grouped by variable type (categorical/continuous)

Outcome	Failure	Birth	*p*-value
Features			
Count	7757 (66.14%)	3971 (33.86%)	
Grades of transferred embryos			
Single good	3693 (47.61%)	1555 (39.16%)	3.73E-129
Both good	2126 (27.41%)	1886 (47.49%)	
1 good and 1 inferior	727 (9.37%)	328 (8.26%)	
Single inferior	839 (10.82%)	116 (2.92%)	
Both inferior	372 (4.8%)	86 (2.17%)	
Number of transferred embryos			
1	4532 (58.42%)	1671 (42.08%)	4.68E-63
2	3225 (41.58%)	2300 (57.92%)	
Ovulation inducing scheme			
Antagonist	3925 (50.6%)	1744 (43.92%)	5.19E-32
Long	3229 (41.63%)	2067 (52.05%)	
Others	603 (7.77%)	160 (4.03%)	
Day of embryo transfer			
2	869 (11.2%)	293 (7.38%)	1.58E-10
3	6882 (88.72%)	3671 (92.45%)	
4	6 (0.08%)	7 (0.18%)	
Type of infertility			
Primary	4400 (56.72%)	2492 (62.75%)	3.85E-10
Secondary infertility	3357 (43.28%)	1479 (37.25%)	
Regular menstrual cycle			
No	1638 (21.12%)	980 (24.68%)	1.29E-05
Yes	6119 (78.88%)	2991 (75.32%)	
College education or above (female)			
No	2308 (29.75%)	1098 (27.65%)	1.86E-02
Yes	5449 (70.25%)	2873 (72.35%)	
Causes of infertility			
Female	4696 (60.54%)	2320 (58.42%)	2.38E-02
Male	1542 (19.88%)	885 (22.29%)	
Both	824 (10.62%)	413 (10.4%)	
Unknown	695 (8.96%)	353 (8.89%)	
Season of embryo transfer			
Spring	1848 (23.82%)	1038 (26.14%)	4.61E-02
Summer	2258 (29.11%)	1118 (28.15%)	
Autumn	2229 (28.74%)	1092 (27.5%)	
Winter	1422 (18.33%)	723 (18.21%)	
ART method			
IVF	3716 (47.91%)	1905 (47.97%)	9.60E-01
ICSI	4041 (52.09%)	2066 (52.03%)	
Number of usable embryos	2.85 (1.92)	3.59 (2.11)	3.04E-96
Number of good-quality embryos	1.71 (1.71)	2.34 (1.93)	1.20E-84
Female age (years)	33.17 (4.65)	31.45 (3.68)	4.02E-79
Number of normal cleavages	3.87 (2.74)	4.8 (2.91)	3.18E-68
Number of normal fertilizations	4.01 (2.8)	4.91 (2.95)	2.53E-64
Male age (years)	34.69 (5.55)	33 (4.69)	1.58E-56
AMH (ng/mL)	3.78 (2.36)	4.36 (2.24)	3.02E-54
Number of mature oocytes	5.48 (3.46)	6.39 (3.52)	7.22E-45
Gn starting dose (IU)	192.58 (64.04)	178.79 (67.76)	2.08E-36
FSH on trigger day (IU/L)	15.25 (4.19)	14.29 (3.89)	3.15E-36
Rate of good-quality embryos (%)	0.54 (0.38)	0.64 (0.34)	1.02E-35
Total number of follicles taken	7.9 (4.15)	8.88 (4.11)	3.01E-34
Length of right ovary (mm)	55.89 (13.63)	59.04 (13)	7.99E-33
Number of follicles on the right ovary	6.79 (4.17)	7.72 (4.23)	1.13E-32
Width of right ovary (mm)	39.29 (10.42)	41.62 (10.19)	1.04E-30
Number of follicles on the left ovary	6.26 (3.87)	7.06 (3.94)	9.64E-28
E2 on trigger day (pg/mL)	2016 (899.49)	2198.77 (895.25)	1.05E-27
Endometrial thickness (mm)	11.15 (2.41)	11.61 (2.31)	8.87E-26
Width of left ovary (mm)	37.95 (10.1)	39.97 (9.85)	2.79E-24
Length of left ovary (mm)	53.92 (13.27)	56.52 (12.76)	4.30E-24
Total Gn days	9.69 (2.68)	10.09 (2.64)	1.54E-17
Number of children	0.13 (0.37)	0.08 (0.3)	2.27E-16
Number of transfer cycles	1.82 (1.1)	1.89 (0.94)	1.10E-15
Number of follicles on Gn starting day	5.93 (8.11)	7.33 (8.95)	1.30E-13
Pelvic effusion (mm)	25.8 (9.02)	27.09 (9.65)	2.09E-13
Baseline T (ng/mL)	0.42 (0.3)	0.44 (0.26)	2.67E-13
Number of follicles on trigger day	5.23 (4.89)	6 (5.25)	6.03E-13
LH on trigger day (IU/L)	2.36 (2.39)	2.09 (1.82)	7.78E-09
Times of miscarriage	0.45 (0.8)	0.38 (0.74)	2.63E-08
Baseline FSH (IU/L)	7.28 (3.14)	6.96 (2.47)	2.85E-08
Rate of normal cleavages (%)	0.74 (0.27)	0.78 (0.23)	1.54E-07
Rate of fertilization (%)	0.74 (0.27)	0.78 (0.23)	6.07E-07
Antral follicle counting	5.93 (8.06)	6.94 (8.78)	2.13E-06
Number of oocyte retrieval cycles	1.68 (1.33)	1.49 (0.87)	3.78E-06
Sperm levels before treatment (b)	16 (4.66)	15.63 (4.64)	1.21E-04
Total Gn dose (IU)	2103.43 (890.68)	2045.89 (841.18)	1.91E-04
P on trigger day (ng/mL)	0.87 (0.39)	0.85 (0.28)	1.70E-03
Sperm levels after treatment (b)	43.95 (6.55)	43.61 (6.85)	2.48E-03
Sperm levels before treatment (a)	13.48 (5.33)	13.16 (5.15)	4.48E-03
Sperm levels after treatment (a)	48.95 (6.45)	49.17 (6.69)	4.95E-03
Baseline E2 (pg/mL)	44.78 (19.02)	43.83 (18.25)	8.51E-03
Infertility years	3.29 (2.57)	3.06 (2.21)	1.03E-02
BMI (kg/m^2^)	22.22 (3.22)	22.1 (3.13)	6.40E-02
Baseline PRL (ng/mL)	13.67 (6.72)	13.8 (6.93)	8.70E-02
Baseline LH (IU/L)	4.52 (2.79)	4.56 (2.72)	1.29E-01
Baseline P (ng/mL)	0.57 (0.22)	0.57 (0.21)	5.75E-01

### Model training and performance

**Fig. 2:**
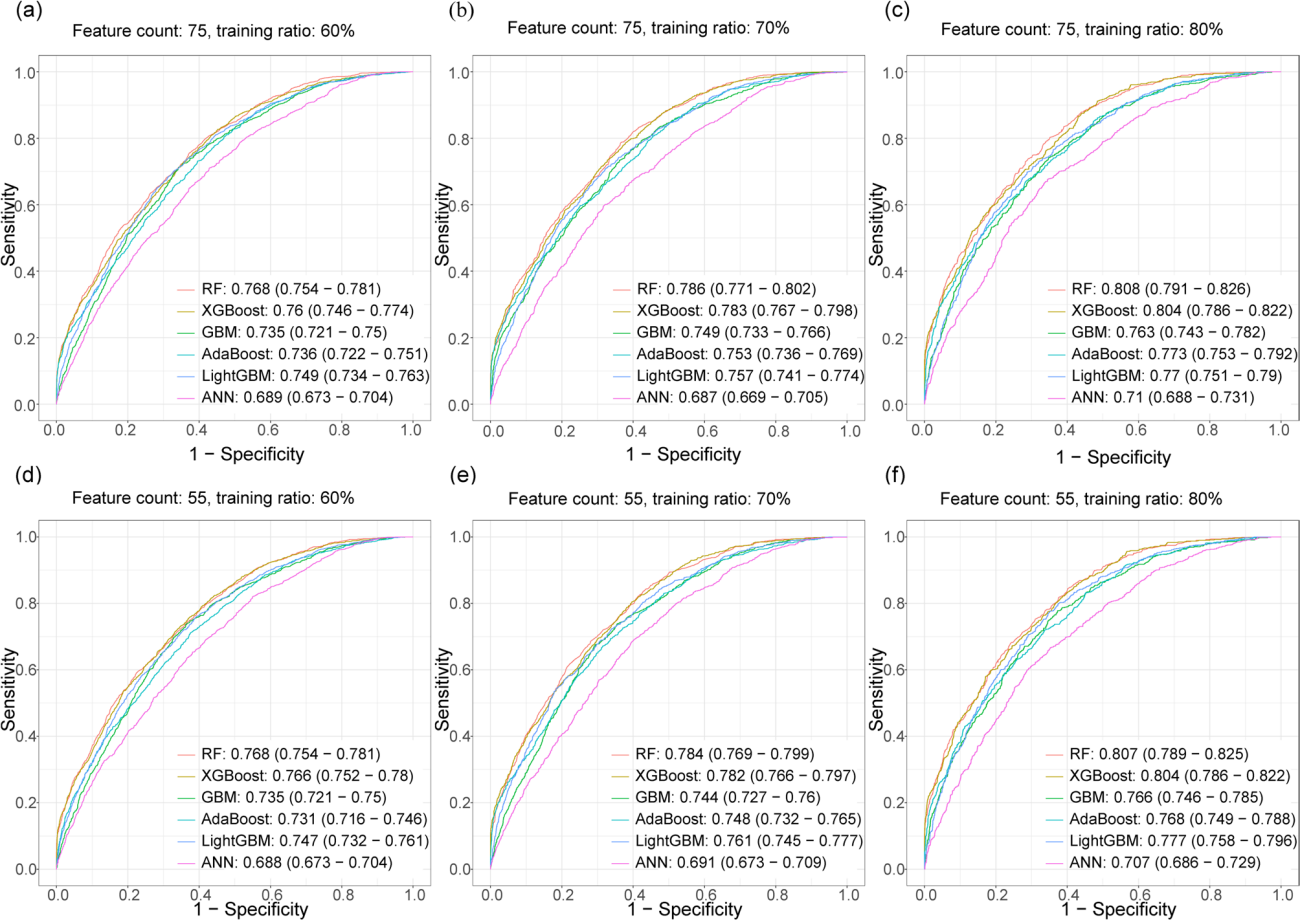
ROC curves comparing the performance of different predictive models under varying feature sets and training-testing allocation ratios. Predictive models include RF, XGBoost, GBM, AdaBoost, LightGBM and ANN. **a**–**c** ROC curves for full feature set models (75 features) trained with **a** 60%, **b** 70%, and **c** 80% data ratios. **d**–**f** Models with refined feature subset (55 features) at matched ratios (**d**: 60%, **e**: 70%, **f**: 80%). All curves report test set performance (remaining 40%/30%/20% unseen data) with AUC values and 95% confidence interval annotated in the lower-right inset

To comprehensively evaluate the performance of various models, the data was partitioned into training and testing sets with three ratios: 60:40, 70:30, and 80:20. Model hyperparameters were optimized using 5-fold cross-validation to maximize the AUC. Classification models were then constructed using the entire training set, initially considering all 75 features. The ROC curves for each model on the testing set are presented in Fig. 2a–c, with the corresponding AUC values and their 95% confidence intervals displayed in the lower right corner. When the training data constituted 80% of the dataset, both random forest and XGBoost achieved AUC values exceeding 0.8, specifically 0.808 (95% CI: 0.791–0.826) for random forest and 0.804 (95% CI: 0.786–0.822) for XGBoost. Across all training proportions, both random forest and XGBoost consistently outperformed other methods in terms of AUC.

To simplify the prediction model, we refined the feature set to 55 variables. In this scenario, both random forest and XGBoost still achieved AUC values exceeding 0.8 on the testing data when using 80% of the data for training (Fig. 2d–f). Similar results were observed across other training data proportions, with performance nearly identical to the models using 75 features. Complementing the discrimination assessment, calibration—measuring agreement between predicted probabilities and observed outcomes—was rigorously evaluated for models trained using the refined 55-feature subset. Calibration curves and Brier scores calculated on the independent test set demonstrated that the random forest model achieved the best calibration among all methods, particularly at the 80% training ratio (Supplementary Fig. S2).

As shown in Table 2, all six models exhibited robust classification performance under three different training-testing splits with the refined predictor set. Among them, RF and XGBoost consistently outperformed others, achieving optimal results not only in AUC but also across supplementary metrics (accuracy, Cohen’s kappa, sensitivity, specificity, etc.) computed at Youden-index-optimized thresholds. RF demonstrated the highest predictive performance, closely followed by XGBoost, whereas ANN yielded the weakest results in this framework.

**Table 2:** Comparative classification performance of six machine learning methods with 55-feature input across three training-testing allocation ratios (60%/70%/80%). Metrics include AUC, accuracy, kappa, sensitivity (Sens), specificity (Spec), precision (Prec), positive/negative likelihood ratios (PLR/NLR), and F1-score, evaluated on testing sets. Bold values highlight the best performance for each metric under the corresponding train ratio

Train ratio	Methods	AUC	Accuracy	Kappa	Sens	Spec	Prec	PLR	NLR	F1
0.8	RF	**0.807**	0.68	**0.379**	0.599	**0.844**	**0.886**	**3.840**	0.475	0.715
	XGBoost	0.804	0.681	0.376	0.612	0.822	0.874	3.438	0.472	0.72
	GBM	0.766	0.677	0.357	0.627	0.778	0.851	2.824	0.479	0.722
	Adaboost	0.768	**0.682**	0.348	**0.664**	0.72	0.828	2.371	**0.467**	**0.737**
	LightGBM	0.777	**0.682**	0.371	0.623	0.801	0.864	3.131	0.471	0.724
	ANN	0.707	0.654	0.287	0.647	0.667	0.797	1.943	0.529	0.714
0.7	RF	**0.784**	0.66	0.348	0.572	**0.836**	**0.875**	**3.488**	0.512	0.692
	XGBoost	0.782	0.671	**0.356**	0.604	0.805	0.861	3.097	0.492	0.71
	GBM	0.744	0.676	0.345	0.645	0.738	0.831	2.462	0.481	0.727
	Adaboost	0.748	0.673	0.327	**0.663**	0.695	0.813	2.174	0.485	0.73
	LightGBM	0.761	**0.682**	0.349	0.661	0.724	0.827	2.395	**0.468**	**0.734**
	ANN	0.691	0.629	0.257	0.597	0.692	0.795	1.938	0.582	0.682
0.6	RF	**0.768**	0.658	0.331	0.593	**0.785**	**0.845**	**2.758**	0.518	0.697
	XGBoost	0.766	**0.672**	**0.344**	0.629	0.756	0.836	2.578	**0.491**	**0.718**
	GBM	0.735	0.67	0.336	**0.634**	0.74	0.828	2.438	0.495	**0.718**
	Adaboost	0.731	0.654	0.302	0.624	0.712	0.81	2.167	0.528	0.705
	LightGBM	0.747	0.66	0.326	0.61	0.758	0.833	2.521	0.515	0.704
	ANN	0.688	0.618	0.242	0.582	0.691	0.788	1.883	0.605	0.669

### Sensitivity analysis for the optimal random forest

To assess the robustness of the optimal random forest model (trained on 80% of data with 55 features), we evaluated its performance across clinically relevant subgroups: female age ($\:\le\:$ 35 vs. >35 years), endometrial thickness ($\:\le\:$ 7 mm, 7–14 mm, > 14 mm), embryo grades, and infertility causes. As summarized in Table 3, the model demonstrated strong discriminatory ability (AUC $\:\ge\:$ 0.80) in most subgroups, particularly for thin endometrium (AUC = 0.856; specificity = 0.977) and advanced maternal age (AUC = 0.814; specificity = 0.916). However, lower performance was observed in patients with high-quality embryo transfers (AUC = 0.703), and sensitivity was consistently low in younger women ($\:\le\:\:$35 years, sensitivity = 0.492). These results suggest that while the model is generally robust, its reliability varies across patient profiles, particularly in favorable prognosis groups.

**Table 3:** Subgroup classification performance of random forest on testing subsets, trained with 55-feature input and 80% training ratios. Metrics include AUC, accuracy, kappa, sensitivity (Sens), specificity (Spec), precision (Prec), positive/negative likelihood ratios (PLR/NLR), and F1-score

Features	Subgroups (*N*)	Live Birth Rate	AUC	Accuracy	Kappa	Sens	Spec	Prec	PLR	NLR	F1
Whole test data (2345)		0.330	0.807	0.680	0.379	0.599	0.844	0.886	3.835	0.476	0.715
Female age (years)	$\:\le\:\:$35 (1739)	0.388	0.779	0.642	0.329	0.492	0.877	0.863	4.005	0.579	0.627
	> 35 (606)	0.165	0.814	0.789	0.366	0.822	0.620	0.916	2.164	0.287	0.867
Endometrial thickness (mm)	$\:\le\:$ 7 (57)	0.105	0.856	0.842	0.447	0.843	0.833	0.977	5.059	0.188	0.905
	(7, 14] (2030)	0.331	0.799	0.679	0.376	0.603	0.835	0.881	3.643	0.476	0.716
	> 14 (258)	0.380	0.852	0.791	0.563	0.806	0.765	0.849	3.435	0.253	0.827
Grades of transferred embryos	Both good (785)	0.459	0.703	0.652	0.295	0.713	0.581	0.667	1.700	0.494	0.689
	1 good and 1 inferior (226)	0.288	0.727	0.779	0.367	0.944	0.369	0.788	1.497	0.151	0.859
	Single good (1039)	0.305	0.818	0.728	0.422	0.717	0.751	0.868	2.879	0.376	0.785
	Both inferior (93)	0.183	0.787	0.688	0.336	0.645	0.882	0.961	5.480	0.403	0.772
	Single inferior (202)	0.079	0.951	0.807	0.374	0.790	1.000	1.000	Inf	0.210	0.883
Causes of infertility	Female (1432)	0.332	0.809	0.714	0.412	0.693	0.758	0.852	2.862	0.405	0.764
	Male (472)	0.314	0.811	0.678	0.383	0.586	0.878	0.913	4.822	0.471	0.714
	Both (242)	0.331	0.833	0.715	0.454	0.611	0.925	0.943	8.148	0.420	0.742
	Unknown (199)	0.362	0.755	0.658	0.357	0.535	0.875	0.883	4.283	0.531	0.667

Additionally, we examined model stability by perturbation of two key features (female age and grades of transferred embryos), with details shown in Supplementary Results. As shown in Supplementary Fig. S3, the model exhibited robustness against perturbations in both features, with relative AUC reductions remaining below 0.5% at clinically plausible noise level.

### Model explanation

**Fig. 3:**
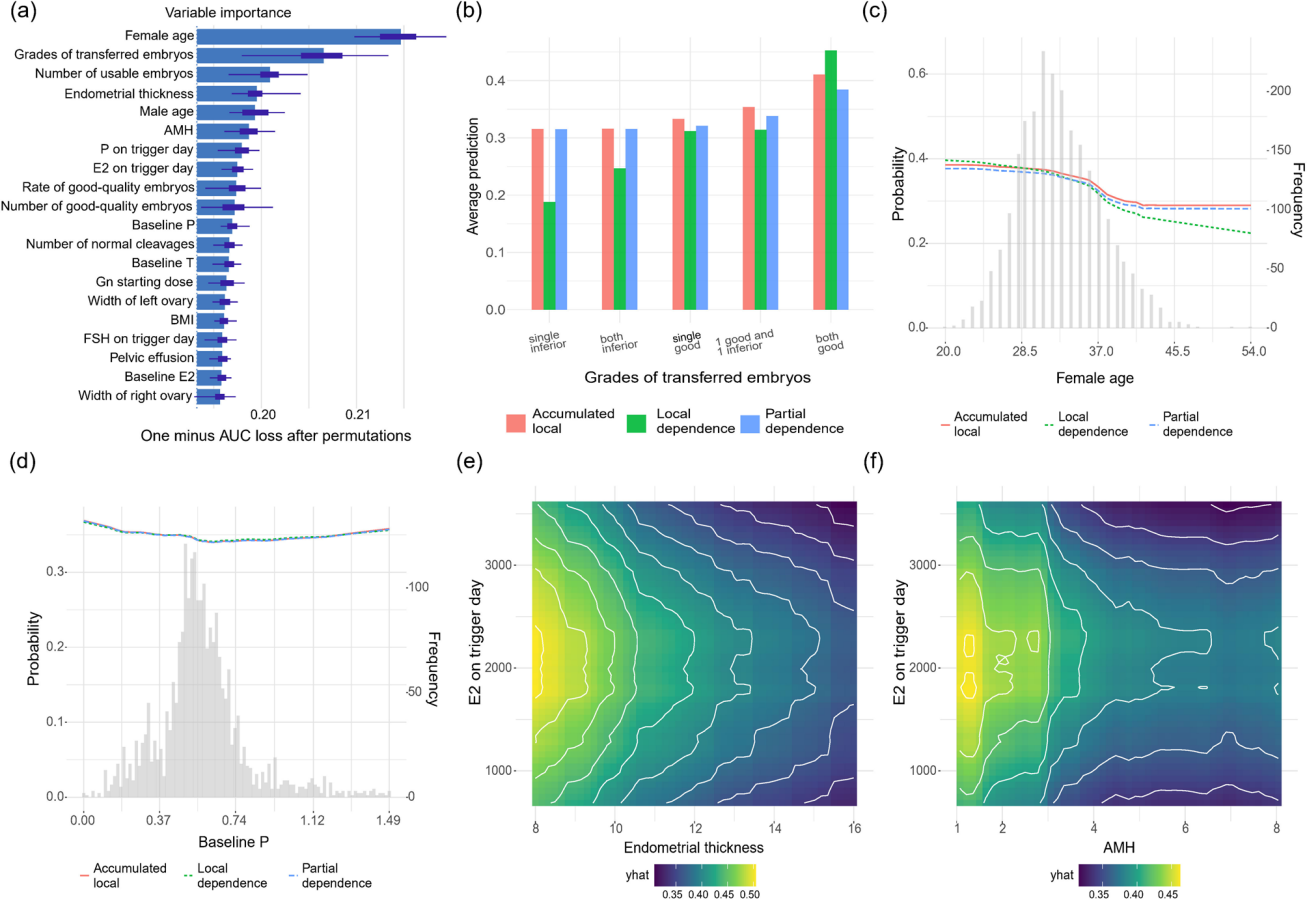
Interpretability analysis of the optimal random forest model (trained on 80% of the dataset) for live birth prediction. **a** Permutation-based importance scores (1− AUC, averaged over 50 repeats) of the top 20 predictive features, ranked by impact. **b**–**d** Influence of individual explanatory predictors on live birth predictions, illustrated through three types of profiles: accumulated local profile, local dependence profile and partial dependence profile. **e-f** Bivariate partial dependence plots demonstrating their joint impact on live birth predictions

To elucidate the mechanisms driving the performance of our optimal random forest model (trained on 80% of the full dataset), we first employed a permutation-based feature importance analysis. This involved iteratively permuting each feature’s values in the testing set (while fixing others) and quantifying the resultant performance degradation using 1−AUC as the importance metric. To mitigate stochastic variability, the permutation process was repeated 50 times; higher values indicated greater feature impact. As shown in Fig. 3a, the top 20 features are ranked by predictive importance. Notably, female age and transferred embryo grades emerged as the two most influential predictors.

In Fig. 3b-d, we illustrate the effects of individual explanatory variables on the prediction of live births by presenting the accumulated local profile, local dependence profile, and partial dependence profile for the grades of transferred embryos, female age and baseline P levels. The transfer of two good embryos is associated with the highest average prediction for live births, followed by scenarios involving one good and one inferior embryos, single good embryo, two inferior embryos, and finally one inferior embryo (Fig. 3b). These findings suggest that appropriately increasing the number of top-quality embryos might enhance live birth potential, particularly in women with previous implantation failure. However, as highlighted in the 2024 Human Reproduction Update [17], careful consideration of medical, economic, social, and psychological implications remains imperative before multi-embryo transfer. Figure 3c shows that the probability of live birth decreases with advancing female age, with a particularly sharp decline around the age of 36. In Fig. 3d, it is evident that the probability of live birth decreases until reaching a plateau at approximately 0.57.

We further illustrate the interaction effects of two variables on the prediction of live births by presenting the two-variable partial dependence profile (Fig. 3e–f). Values are positioned between the 5th and 95th percentiles, with a stronger yellow color indicating a better prediction for live births. Figure 3e shows the interaction effects of estradiol (E2) on the trigger day and endometrial thickness. As endometrial thickness increases from 8 to 16 mm, the probability of live birth consistently decreases across all values of E2 on the trigger day. The optimal E2 level on the trigger day is approximately 2,000 pg/mL. Figure 3f reveals a similar pattern, where an AMH value between 1 ng/mL and 2 ng/mL and an E2 level of around 2,000 pg/mL correspond to the strongest prediction for live births.

### Web implementation

**Fig. 4:**
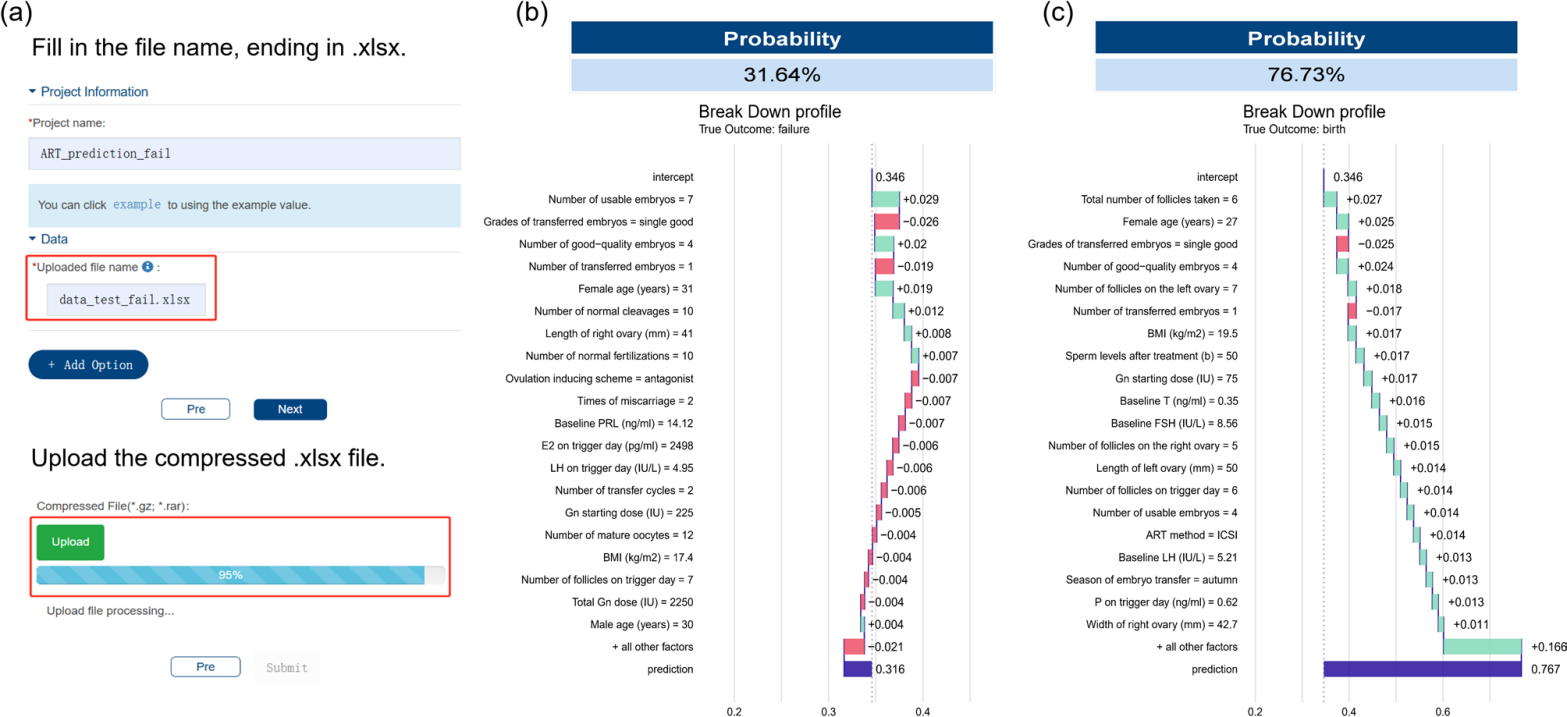
Clinical implementation and case-level interpretation of the random forest prediction model. **a** Interface of the web-based prediction tool (https://bmap.sjtu.edu.cn/platform/details/92), allowing clinicians to upload patient data and receive live birth chance estimates along with explanatory feature insights. **b**–**c** Instance-specific explanation using breakdown profiles for **b** a failed outcome and **c** a successful live birth outcome

To facilitate clinical translation, we deployed the optimal random forest model as a web-based tool (Fig. 4a) integrated within the Biomedical Data Analysis Platform (BMAP) [18]. Clinicians can upload patient data to predict live birth chance following fresh embryo transfer and identify influential features. The platform operates through internet access, with all computational processing executed on cloud servers, thereby eliminating the need for local computing infrastructure. To accommodate potential limitations in internet connectivity, we have incorporated a downloadable pre-processed analysis module that supports offline functionality. Comprehensive documentation is available at https://bmap.sjtu.edu.cn/platform/details/92 to ensure seamless implementation and adoption.

In our testing dataset, we selected cases representing both successful and unsuccessful live births. By uploading the relevant testing data, we obtained the results illustrated in Fig. 4b and c, which include detailed profiles that elucidate the prediction model’s mechanisms from an instance-specific perspective. In Fig. 4b, the top three contributors to the live birth prediction for this individual are the number of usable embryos, the grades of transferred embryos, and the number of high-quality embryos. A detailed analysis reveals that a small number of transferred embryos and the transfer of only one good embryo decrease the probability for live birth, compounded by several weak yet negative factors contributing to the unsuccessful outcome. In contrast, Fig. 4c indicates that most features exert a positive influence, despite the same low values for the number of transferred embryos and the transfer of only one high-quality embryo.

## Discussion

As previously reported, the miscarriage rate following IVF is notably high [4, 19]. Generally, physicians predict pregnancy outcomes after embryo transfer by assessing whether the patient’s examination indices fall within the normal range [5]. Although these monitoring tests often uncover irreversible defects, they are frequently detected too late to prevent harm, especially for infertile women. In contrast to the diagnostic approach, our RF model is more insightful, efficient, and accurate. Compared to prior studies [20, 21], our model demonstrates superior predictive accuracy and utilizes a substantially larger clinical dataset (A total of 11,728 ART records), achieving a predictive precision of 80.8%.

Among the 75 features, the top six contributors to success in ARTs were female age, grades of transferred embryos, number of usable embryos, endometrial thickness, male age, and AMH. It is well established that advanced female age is associated with lower ART success rates due to diminished ovarian reserve, reduced oocyte quality, and an increased risk of chromosomal abnormalities [22]. Female age has been identified as a crucial predictor in nearly all related studies [23–25]. Clinically, for patients aged over 35 years, strict monitoring of trigger-day estradiol and progesterone levels is required. If values exceed thresholds, cancellation of fresh embryo transfer and switching to a freeze-all strategy is recommended to avoid cycle wastage. Furthermore, transferring two or at least one high-quality embryo significantly increases the likelihood of live birth, consistent with prior research findings [26, 27]. The number of usable embryos retrieved also markedly influences the live birth rate. Patients undergoing ARTs can preserve a greater number of embryos before transfer, rather than rushing into a single cycle. Evidence suggests that frozen blastocyst transfers may enhance live birth rates, aligning with prior research [28]. Our research also indicates that the optimal endometrial thickness for transfer is between 8 and 8.5 mm with the optimal E2 level on the trigger day at approximately 2,000 pg/mL. In fresh embryo transfer cycles, elevated E2 level has been associated with improved live birth up to a threshold at approximately 2,000–2,999 pg/mL, beyond which benefits plateau [29]. This threshold can guide cycle cancellation decisions for low responders (< 1,000 pg/mL) and stimulation protocol adjustments in subsequent cycles.

Endometrial thickness correlates with E2 levels on the trigger day. Females with an endometrial thickness of less than 8 mm and abnormal E2 levels on the trigger day may experience negative outcomes following ARTs. Simultaneously, the value of endometrial pattern and blood flow must be emphasized. The positive correlation between AMH levels and live birth rates observed in our study aligns with prior findings [30–32]. AMH serves as an indicator of the ovarian follicular pool and has long been used as a marker of ovarian reserve [33]. In fact, AMH is closely associated with embryo quality and the number of usable embryos [34, 35]. Fresh embryo transfer may be a better choice for women with low prognosis in terms of live birth rate compared with a freeze-all strategy. The treatment strategies that prevent fresh embryo transfers, such as accumulating embryos with back-to-back cycles or performing routine preimplantation genetic testing for aneuploidy, warrant further studies in women with a low prognosis [36]. This also confirms the importance of AMH, consistent with our study’s findings. In addition to these variables, other hormonal levels, such as progesterone (P), testosterone (T), and follicle-stimulating hormone (FSH) on the trigger day, are also related to ART outcomes and exhibit significant predictive value.

Overall, this study presents a novel method for predicting pregnancy outcomes in patients undergoing ARTs prior to embryo transfer, demonstrating satisfactory predictive performance. The non-invasive blood test, combined with basic clinical information, renders the approach cost-effective for clinical application. Our model specifically addresses fresh embryo transfers, as we posit that distinct prediction models are required for fresh versus frozen cycles due to fundamental differences in their underlying biological mechanisms and clinical variables. However, several limitations should be noted when applying this model. First, although the sample size is large, the single-center nature of this study restricts the generalizability of the findings. Additional datasets from multiple centers should be recruited to enhance the external validation of our prediction model. Second, while our study highlighted the significant role of embryo quality in the prediction model, ranked just below female age, the assessment of embryo quality relied on visual inspection of morphology, which is subjective and dependent on the experience of practitioners [37]. Employing AI for embryo morphological grading could enhance the objectivity of our prediction model. Third, the model’s current framework is specific to cleavage-stage fresh embryo transfers, limiting its immediate applicability to frozen cycles and blastocyst transfers. Fourth, in clinical application, it is crucial to recognize that model outputs should serve as decision-support tools rather than definitive predictors. This is particularly important for probability estimates near critical decision thresholds, where patient-specific factors—including age and prior treatment history—may significantly influence risk-benefit considerations. Ethical implementation requires comprehensive informed consent processes that emphasize patient autonomy, clearly communicate the probabilistic nature of predictions, and maintain clinician judgment as central to decision-making.

## Conclusions

This study applied six different machine learning models to predict live birth following fresh blastocyst transfer in ARTs. The random forest (RF) model demonstrated the best predictive performance, achieving an AUC value of 0.808. Key predictors identified included female age, grades of transferred embryos, number of usable embryos, endometrial thickness, male age, and AMH levels. With the assistance of machine learning-driven prediction models, clinicians can provide more accurate recommendations and estimates to patients before embryo transfer, thereby enhancing the success rate of ARTs.

## Supplementary Information

Below is the link to the electronic supplementary material.

Supplementary Material 1

Supplementary Material 2

## Data Availability

All data are available in the manuscript or the supplementary materials. All original code and implementation details are available in a public GitHub repository hosted at https://github.com/Cinbo-Wang/ART_prediction. Any additional information required to reanalyze the data reported in this paper is available from the lead contact upon request.

## Abbreviations

ARTs: Assisted reproductive technologies
RF: Random forest
XGBoost: eXtreme gradient boosting
GBM: Gradient boosting machines
AdaBoost: Adaptive boosting
LightGBM: Light gradient boosting machine
ANN: Artificial neural network
AUC: Area under the receiver operating characteristic curve
IVF: In vitro fertilization
ICSI: Intracytoplasmic sperm injection
BMI: Body mass index
AMH: Anti-müllerian hormone
AI: Artificial intelligence
PD: Partial dependence
LD: Local dependence
AL: Accumulated local
Sens: Sensitivity
Spec: Specificity
Prec: Precision
PLR/NLR: Positive/negative likelihood ratios
E2: Estradiol
BMAP: Biomedical data analysis platform
P: Progesterone
T: Testosterone
FSH: Follicle-stimulating hormone

## References

[1] Al-Ali H, Baig A, Alkhanjari RR, Murtaza ZF, Alhajeri MM, Elbahrawi R, Abdukadir A, Bhamidimarri PM, Kashir J, Hamdan H. Septins as key players in spermatogenesis, fertilisation and pre-implantation embryogenic cytoplasmic dynamics. Cell Commun Signal. 2024;22:523. 39468561 10.1186/s12964-024-01889-z PMC11514797

[2] Inhorn MC, Patrizio P. Infertility around the globe: new thinking on gender, reproductive technologies and global movements in the 21st century. Hum Reprod Update. 2015;21:411–26. 25801630 10.1093/humupd/dmv016

[3] Kjaer ASL, Vestager ML, Jensen RB, Pinborg A. Healthy aging in individuals born after assisted reproductive technology is a research area for the future. Nat Aging. 2024;4:1663–6. 39672889 10.1038/s43587-024-00721-0

[4] Chien CW, Tang YA, Jeng SL, Pan HA, Sun HS. Blastocyst telomere length predicts successful implantation after frozen-thawed embryo transfer. Hum Reprod Open. 2024;hoae012. 10.1093/hropen/hoae012 PMC10955253 38515829

[5] Fu K, Li Y, Lv H, Wu W, Song J, Xu J. Development of a model predicting the outcome of in vitro fertilization cycles by a robust decision tree method. Front Endocrinol (Lausanne). 2022;13:877518. 36093079 10.3389/fendo.2022.877518 PMC9449728

[6] Al-Lamee H, Stone K, Powell SG, Wyatt J, Drakeley AJ, Hapangama DK, Tempest N. Endometrial compaction to predict pregnancy outcomes in patients undergoing assisted reproductive technologies: a systematic review and meta-analysis. Hum Reprod Open. 2024;hoae040. 10.1093/hropen/hoae040 PMC11239225 38993630

[7] Liu X, Chen Z, Ji Y. Construction of the machine learning-based live birth prediction models for the first in vitro fertilization pregnant women. BMC Pregnancy Childbirth. 2023;23:476. 37370040 10.1186/s12884-023-05775-3 PMC10294395

[8] Iliodromiti S, Kelsey TW, Wu O, Anderson RA, Nelson SM. The predictive accuracy of anti-Müllerian hormone for live birth after assisted conception: a systematic review and meta-analysis of the literature. Hum Reprod Update. 2014;20:560–70. 24532220 10.1093/humupd/dmu003

[9] Sermondade N, Huberlant S, Bourhis-Lefebvre V, Arbo E, Gallot V, Colombani M, Fréour T. Female obesity is negatively associated with live birth rate following IVF: a systematic review and meta-analysis. Hum Reprod Update. 2019;25:439–51. 30941397 10.1093/humupd/dmz011

[10] Wong KM, van Wely M, Verhoeve HR, Kaaijk EM, Mol F, van der Veen F, Repping S, Mastenbroek S. Transfer of fresh or frozen embryos: a randomised controlled trial. Hum Reprod. 2021;36:998–1006. 33734369 10.1093/humrep/deaa305 PMC7970725

[11] Sun L, Li J, Zeng S, Luo Q, Miao H, Liang Y, Cheng L, Sun Z, Tai WH, Han Y, et al. Artificial intelligence system for outcome evaluations of human in vitro fertilization-derived embryos. Chin Med J (Engl). 2024;137:1939–49. 38997251 10.1097/CM9.0000000000003162 PMC11332789

[12] Beam AL, Kohane IS. Big data and machine learning in health care. JAMA. 2018;319:1317–8. 29532063 10.1001/jama.2017.18391

[13] Liu L, Jiao Y, Li X, Ouyang Y, Shi D. Machine learning algorithms to predict early pregnancy loss after in vitro fertilization-embryo transfer with fetal heart rate as a strong predictor. Comput Methods Programs Biomed. 2020;196:105624. 32623348 10.1016/j.cmpb.2020.105624

[14] Curchoe CL, Bormann CL. Artificial intelligence and machine learning for human reproduction and embryology presented at ASRM and ESHRE 2018. J Assist Reprod Genet. 2019;36:591–600. 30690654 10.1007/s10815-019-01408-x PMC6504989

[15] Si B. Explanatory model analysis: explore, explain, and examine predictive models. J Qual Technol. 2021;54:486–486.

[16] Stekhoven DJ, Bühlmann P. MissForest—non-parametric missing value imputation for mixed-type data. Bioinformatics. 2011;28:112–8. 22039212 10.1093/bioinformatics/btr597

[17] Alteri A, Arroyo G, Baccino G, Craciunas L, De Geyter C, Ebner T, Koleva M, Kordic K, McHeik S, Mertes H, et al. ESHRE guideline: number of embryos to transfer during IVF/ICSI†. Hum Reprod. 2024;39:647–57. 38364208 10.1093/humrep/deae010 PMC10988112

[18] Ren Y, Cheng Z, Li L, Zhang Y, Dai F, Deng L, Wu Y, Gu J, Lin Q, Wang X et al. BMAP: a comprehensive and reproducible biomedical data analysis platform. bioRxiv 2024;603507.

[19] Sunderam S, Kissin DM, Zhang Y, Jewett A, Boulet SL, Warner L, Kroelinger CD, Barfield WD. Assisted Reproductive technology surveillance—United States, 2018. MMWR Surveill Summ 2022;71:1–19. 10.15585/mmwr.ss7104a1 PMC8865855 35176012

[20] Barnett-Itzhaki Z, Elbaz M, Butterman R, Amar D, Amitay M, Racowsky C, Orvieto R, Hauser R, Baccarelli AA, Machtinger R. Machine learning vs. classic statistics for the prediction of IVF outcomes. J Assist Reprod Genet. 2020;37:2405–12. 32783138 10.1007/s10815-020-01908-1 PMC7550518

[21] Wang CW, Kuo CY, Chen CH, Hsieh YH, Su EC. Predicting clinical pregnancy using clinical features and machine learning algorithms in in vitro fertilization. PLoS ONE. 2022;17:e0267554. 35675328 10.1371/journal.pone.0267554 PMC9176781

[22] Fu W, Cui Q, Bu Z, Shi H, Yang Q, Hu L. Elevated sperm DNA fragmentation is correlated with an increased chromosomal aneuploidy rate of miscarried conceptus in women of advanced age undergoing fresh embryo transfer cycle. Front Endocrinol (Lausanne). 2024;15:1289763. 38650716 10.3389/fendo.2024.1289763 PMC11033384

[23] Liu Z, Zhang H, Xiong F, Huang X, Yu S, Sun Q, Diao L, Li Z, Wu Y, Zeng Y, Huang C. Prediction of clinical pregnancy outcome after single fresh blastocyst transfer during in vitro fertilization: an ensemble learning perspective. Hum Fertil (Camb). 2024;27:2422918. 39523806 10.1080/14647273.2024.2422918

[24] Dias CMF, Furlan SMP, Ferriani RA, Navarro P. Serum progesterone measurement on the day of fresh embryo transfer and its correlation with pregnancy success rates: a prospective analysis. Clin (Sao Paulo). 2024;79:100511. 10.1016/j.clinsp.2024.100511 PMC11736334 39388739

[25] Liu L, Liu B, Liao M, Gan Q, Huang Q, Yang Y. Identifying key predictive features for live birth rate in advanced maternal age patients undergoing single vitrified-warmed blastocyst transfer. Reprod Biol Endocrinol. 2024;22:120. 39375693 10.1186/s12958-024-01295-7 PMC11457422

[26] Motato Y, de los Santos MJ, Escriba MJ, Ruiz BA, Remohí J, Meseguer M. Morphokinetic analysis and embryonic prediction for blastocyst formation through an integrated time-lapse system. Fertil Steril. 2016;105:376–e384379. 26598211 10.1016/j.fertnstert.2015.11.001

[27] Li X, Zeng Y, Zhu L, Yang Z, Luo Y, Jia JL. The association between pregnancy outcomes and frozen-thawed embryo transfer cycles based on D3 cell count in high-quality blastocysts. Front Endocrinol (Lausanne). 2024;15:1464313. 39493775 10.3389/fendo.2024.1464313 PMC11527635

[28] Wei D, Liu JY, Sun Y, Shi Y, Zhang B, Liu JQ, Tan J, Liang X, Cao Y, Wang Z, et al. Frozen versus fresh single blastocyst transfer in ovulatory women: a multicentre, randomised controlled trial. Lancet. 2019;393:1310–8. 30827784 10.1016/S0140-6736(18)32843-5

[29] Lersten IL, Grau L, Jahandideh S, Devine K, Zalles L, Plosker SM, Imudia AN, Hoyos LR, Uhler ML, Homer M, et al. High estradiol levels in fresh embryo transfer cycles are not associated with detrimental impact on birth outcomes. J Assist Reprod Genet. 2024;41:893–902. 38600428 10.1007/s10815-024-03062-4 PMC11052734

[30] Chinè A, Reschini M, Fornelli G, Basili L, Busnelli A, Viganò P, Muzii L, Somigliana E. Low ovarian reserve and risk of miscarriage in pregnancies derived from assisted reproductive technology. Hum Reprod Open 2023;2023:hoad026. 10.1093/hropen/hoad026 PMC10243845 37287447

[31] Sellami I, Barbotin AL, Bernard V, Robin G, Catteau-Jonard S, Sonigo C, Peigné M. Anti-Mullerian hormone assessment in assisted reproductive technique outcome and natural conception. Semin Reprod Med. 2024;42:25–33. 39025077 10.1055/s-0044-1787273

[32] Peigné M, Bernard V, Dijols L, Creux H, Robin G, Hocké C, Grynberg M, Dewailly D, Sonigo C. Using serum anti-Müllerian hormone levels to predict the chance of live birth after spontaneous or assisted conception: a systematic review and meta-analysis. Hum Reprod. 2023;38:1789–806. 37475164 10.1093/humrep/dead147

[33] Panay N, Anderson RA, Bennie A, Cedars M, Davies M, Ee C, Gravholt CH, Kalantaridou S, Kallen A, Kim KQ et al. Evidence-based guideline: premature ovarian insufficiency(). Hum Reprod Open 2024;2024:hoae065. 10.1093/hropen/hoae065 PMC11631070 39660328

[34] Han Z, Liu J, Liang T, Yin J, Wei J, Zeng Q, Cao W, Liu C, Sun S. Exposure to ambient particulate matter and ovarian reserve impairment among reproductive age women in China. J Hazard Mater. 2024;480:136212. 39454334 10.1016/j.jhazmat.2024.136212

[35] Arce JC, La Marca A, Mirner Klein B, Nyboe Andersen A, Fleming R. Antimüllerian hormone in gonadotropin releasing-hormone antagonist cycles: prediction of ovarian response and cumulative treatment outcome in good-prognosis patients. Fertil Steril. 2013;99:1644–53. 23394782 10.1016/j.fertnstert.2012.12.048

[36] Wei D, Sun Y, Zhao H, Yan J, Zhou H, Gong F, Zhang A, Wang Z, Jin L, Bao H, et al. Frozen versus fresh embryo transfer in women with low prognosis for in vitro fertilisation treatment: pragmatic, multicentre, randomised controlled trial. BMJ. 2025;388:e081474. 39880462 10.1136/bmj-2024-081474 PMC11778674

[37] Storr A, Venetis CA, Cooke S, Kilani S, Ledger W. Inter-observer and intra-observer agreement between embryologists during selection of a single day 5 embryo for transfer: a multicenter study. Hum Reprod. 2017;32:307–14. 28031323 10.1093/humrep/dew330

