# Correction: Predictive models for live birth outcomes following fresh embryo transfer in assisted reproductive technologies using machine learning

**DOI:** 10.1186/s12967-025-07248-x

**Published:** 2025-10-07

**Authors:** Shengnan Wu, Xinbo Wang, Yuechen Liu, Yongyong Ren, Mei Zhao, Haitao Song, Hao Shen, Yueting Wu, Zhiyun Wei, Hui Lu, Kunming Li

**Affiliations:** 1 Department of Integrated Traditional Chinese Medicine (TCM) & Western Medicine, Shanghai Key Laboratory of Maternal Fetal Medicine, Shanghai Institute of Maternal- Fetal Medicine and Gynecologic Oncology, Clinical and Translational Research Center, Shanghai First Maternity and Infant Hospital, School of Medicine, Tongji University, Shanghai, 201204 China; 2 Department of Bioinformatics and Biostatistics, School of Life Sciences and Biotechnology, Shanghai Jiao Tong University, Shanghai, 200240 China; 3 SJTU-Yale Joint Center for Biostatistics and Data Science, Technical Center for Digital Medicine, National Center for Translational Medicine, Shanghai Jiao Tong University, Shanghai, 200240 China; 4 Institute of Bioinformatics, Shanghai Academy of Experimental Medicine, Shanghai, 200240 China; 5 Shanghai Children’s Hospital, School of Medicine, Shanghai Jiao Tong University, Shanghai, 200062 China; 6 Center for Reproductive Medicine, Shanghai First Maternity and Infant Hospital, School of Medicine, Tongji University, Shanghai, 201204 China; 7 Shanghai Artificial Intelligence Research Institute, Shanghai, 200240 China; 8 XiangFu Laboratory, Jiashan, 314102 China; 9 Shanghai Key Lab of Reproduction and Development, Shanghai Key Lab of Female Reproductive Endocrine Related Diseases, Obstetrics & Gynecology Hospital of Fudan University, Shanghai, 200433 China; 10 Department of Reproductive Medicine, Shanghai Tenth People’s Hospital, Tongji University School of Medicine, Shanghai, 200072 China

**Correction to: *J Transl Med*** **23, 1004 (2025).**

10.1186/s12967-025-07045-6

Following publication of the original article [1], the authors reported an error in the authorship. Equal contribution for the authors Shengnan Wu and Xinbo Wang was inadvertently omitted. This has now been corrected.

The original article [1] has been updated.
