# Supplementary material for: Predictive models for live birth outcomes following fresh embryo transfer in assisted reproductive technologies using machine learning
Source: J Transl Med. 2025 Sep 24;23:1004. doi: 10.1186/s12967-025-07045-6 (PMC12462326; doi:10.1186/s12967-025-07045-6)
Supplement: Supplementary file 2 — Supplementary Material 2 [file 12967_2025_7045_MOESM2_ESM.docx]

Supplementary material for

**Predictive Models for Live Birth Outcomes Following Fresh Embryo Transfer in Assisted Reproductive Technologies Using Machine Learning**

Shengnan Wu^1,11^, Xinbo Wang^2,3,11^, Yuechen Liu^3^, Yongyong Ren^3,4,5^, Mei Zhao^6^, Haitao Song^3,7^, Hao Shen^7,8^, Yueting Wu^2^, Zhiyun Wei^1,9*^, Hui Lu^2,3,^^5*^, Kunming Li^1,6,10*^

^1^ Department of Integrated Traditional Chinese Medicine (TCM) & Western Medicine, Shanghai Key Laboratory of Maternal Fetal Medicine, Shanghai Institute of Maternal-Fetal Medicine and Gynecologic Oncology, Clinical and Translational Research Center, Shanghai First Maternity and Infant Hospital, School of Medicine, Tongji University, Shanghai 201204, China

^2^ State Key Lab of Microbial Metabolism, Joint International Research Laboratory of Metabolic Developmental Sciences, Department of Bioinformatics and Biostatistics, School of Life Sciences and Biotechnology, Shanghai Jiao Tong University, Shanghai 200240, China

^3^ SJTU-Yale Joint Center for Biostatistics and Data Science, Technical Center for Digital Medicine, National Center for Translational Medicine, Shanghai Jiao Tong University, Shanghai 200240, China

^4^ Institute of Bioinformatics, Shanghai Academy of Experimental Medicine, Shanghai 200240, China

^5^ Shanghai Children's Hospital, School of Medicine, Shanghai Jiao Tong University, Shanghai 200062, China

^6^ Center for Reproductive Medicine, Shanghai First Maternity and Infant Hospital, School of Medicine, Tongji University, Shanghai, 201204, China

^7^ Shanghai Artificial Intelligence Research Institute, Shanghai 200240, China

^8^ XiangFu Laboratory, Jiashan, 314102, China

^9^ Obstetrics & Gynecology Hospital of Fudan University, Shanghai Key Lab of Reproduction and Development, Shanghai Key Lab of Female Reproductive Endocrine Related Diseases, 200433, Shanghai, China

^10^ Department of Reproductive Medicine, Shanghai Tenth People’s Hospital, Tongji University School of Medicine, Shanghai 200072, China

^11^ These authors contribute equally

* Correspondence: drlikunming@qq.com (K.L.); huilu@sjtu.edu.cn (H.L.); zhiyun_wei@163.com (Z.W.)

**METHODS**

**Hyper-parameter optimization**

In the main text, we considered six machine learning models, and used grid search approach for searching the optimal hyper-parameters. Here, we give the key hyper-parameters for each method.

Random forest (RF): number of variables randomly sampled as candidates at each split (mtry = 4, 5, …, 10), number of trees to grow (ntree = 200, 300, …, 1200).

XGBoost: the max number of iterations (nrounds = 1800, 2000, 2200, 2400), maximum depth of the tree (max_depth = 9, 12, 15, 18), step size of each boosting step (eta = 0.01, 0.05), minimum loss reduction required to make a further partition on a leaf node of the tree (gamma = 0.01, 0.05), subsample ratio of columns when constructing each tree (colsample_bytree = 0.4, 0.5, 0.6).

LightGBM: The hyper parameter grids are generated using grid_max_entropy() in the R package *dials* (version 1.2.1)*,* with total number of parameter value combinations to be 200. The number of trees contained in a boosted ensemble (trees $\in[100, 1000]$), the number of predictors that will be randomly sampled at each split when creating tree models (mtry $\in[3, 8]$), the maximum depth of the tree (tree_depth $\in[3, 15]$), the minimum number of data points in a node that is required for the node to be split further (min_n $\in[10, 40]$).

Gradient boosting model (GBM): The maximum depth of variable interactions (interaction.depth = 7, 9, 11, 13), the total number of trees to fit (n.trees = 300, 400, …, 2000), the learning rate (shrinkage = 0.01, 0.05, 0.1), the minimum number of observations in the trees terminal nodes (n.minobsinnode = 10).

AdaBoost: The number of iterations for which boosting is run (mfinal = 500, 600, …, 3000), maximum depth of the tree (maxdepth = 3, 4, 5, 6).

ANN: The structure of ANN used in the main text is shown in Fig. S1. The learning rate = 1e-4, 1e-3, 1e-2, 1e-1, batch size = 32, 64, 128. We used the AdamW() optimizer with weight decay = 1e-3, 1e-4.

**Results**

**Calibration Analysis**

Complementing the discrimination assessment, calibration—measuring agreement between predicted probabilities and observed outcomes—was rigorously evaluated for models trained using the refined 55-feature subset. To address this point, we have conducted rigorous calibration analyses:

Calibration curves and Brier scores were systematically evaluated across models trained at different ratios (60%, 70%, 80%), using the 55-feature subset on the independent test set. Fig. S2 illustrates these results, demonstrating that:

1. The Random Forest model achieved the best calibration among all compared methods (including XGBoost and others), with optimal performance at the 80% training ratio (Brier score = 0.171).

2. The calibration curves show moderate alignment between predicted probabilities and observed event rates, though some deviations are noted in lower-risk ranges.

While the Brier score of 0.171 indicates room for improvement in probability estimation, the model maintains acceptable calibration for population-level risk stratification, complemented by its strong discrimination (AUC=0.807).

**Sensitivity analysis for the optimal random forest**

To assess the model’s stability, we additionally conducted comprehensive sensitivity tests following these methodological approaches:

1. Continuous Variable Perturbation (Female age):

- Augment the original feature by introducing Gaussian noise with standard deviations ranging from 10–50% of the variable's original standard deviation (κ = {0.1, 0.2, ..., 0.5}).
- Performed 100 Monte Carlo iterations per noise level.

1. Categorical Variable Perturbation (Grades of transferred embryos):

- Implemented random mislabeling of 2–20% of samples ($\eta$∈{0.02, 0.04, ..., 0.20}), with corruption following a uniform distribution across classes.
- Performed 100 Monte Carlo iterations per misclassification level.

1. Quantitative Evaluation: For our best-performing random forest model (trained on 80% data, 55 features), we calculated:

- Mean post-perturbation AUC.
- Mean Relative AUC change: (AUC_original - AUC_perturbed) / AUC_original × 100%.

Key findings are presented in Fig. S3. The model exhibited resilience against perturbations in female age (AUC decline <0.5% at $\kappa=0.5$). And the performance remained robust after grades of transferred embryos mislabeling (AUC decline <0.5% at $\eta=0.20$). Both these results validate our model's stability, with relative AUC reductions remaining below 0.5% at clinically plausible noise level.

**Interpretability Methods**

In this study, we used three interpretability methods (Partial Dependence [PD], Accumulated Local [AL] profiles, and Local Dependence [LD]). As the mean of ceteris-paribus (CP) profiles, PD plots illustrate how predictions change when a single variable is modified while others remain fixed (Friedman 2001). They are intuitive under independence and additivity but may yield misleading interpretations for correlated variables due to reliance on marginal distributions. While, LD accounts for conditional distributions, yet if variables are correlated, LD may inadvertently capture effects of other predictors (Apley & Zhu 2020). Proposed by Apley & Zhu (2020), AL profiles mitigate collinearity biases by averaging local effects, providing robust summaries even for correlated variables in additive models.

**TABLES**

**Table S2** Comparison of three interpretability techniques.

| Method​​ | ​​Key Concept​​ | ​​Advantage​​ | ​​Limitation​ |
| --- | --- | --- | --- |
| Partial Dependence (PD) Profiles​​ | Average prediction changes when modifying one variable while ​**​others remain fixed​**​ | Intuitive visualization under feature independence and model additivity | Misleading with strong correlated features |
| ​​Local Dependence (LD) Profiles​​ | Effects based on ​**​conditional distributions​**​ of features | More realistic than PD for correlated variables | May inadvertently capture effects of correlated predictors |
| Accumulated Local (AL) Profiles​​ | Average ​**​local effects​**​ while accounting for feature collinearity | Robust interpretation for correlated features in additive models | Primarily designed for additive model structures |

**FIGURES**


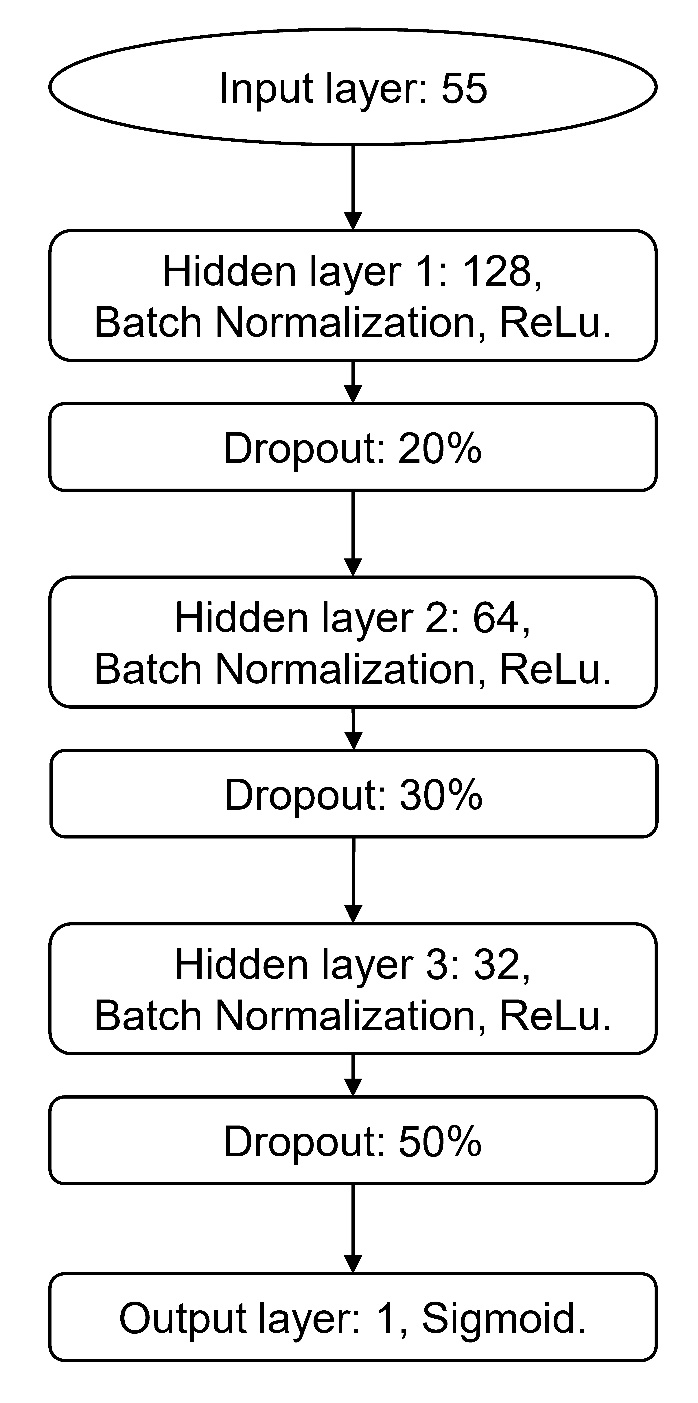


**Fig. S1** Structure of ANN used in the main text.


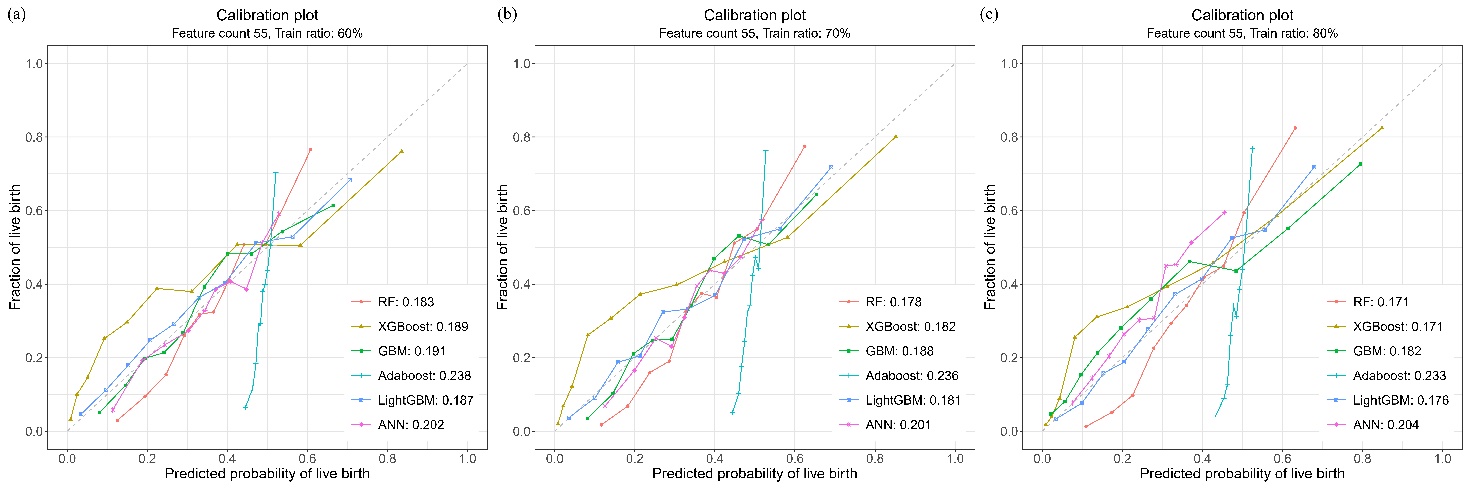


**Fig. S2** Calibration curves comparing the performance of different predictive models under varying training-testing allocation ratios using 55 features. Predictive models include Random Forest (RF), XGBoost, Gradient Boosting Model (GBM), AdaBoost, LightGBM and ANN. (a-c) Calibration curves for 55 feature set models trained with 60% (a), 70% (b), and 80% (c) data ratios. All curves report performance on the test data (remaining 40%/30%/20% unseen data) with Brier score annotated in the lower-right inset.


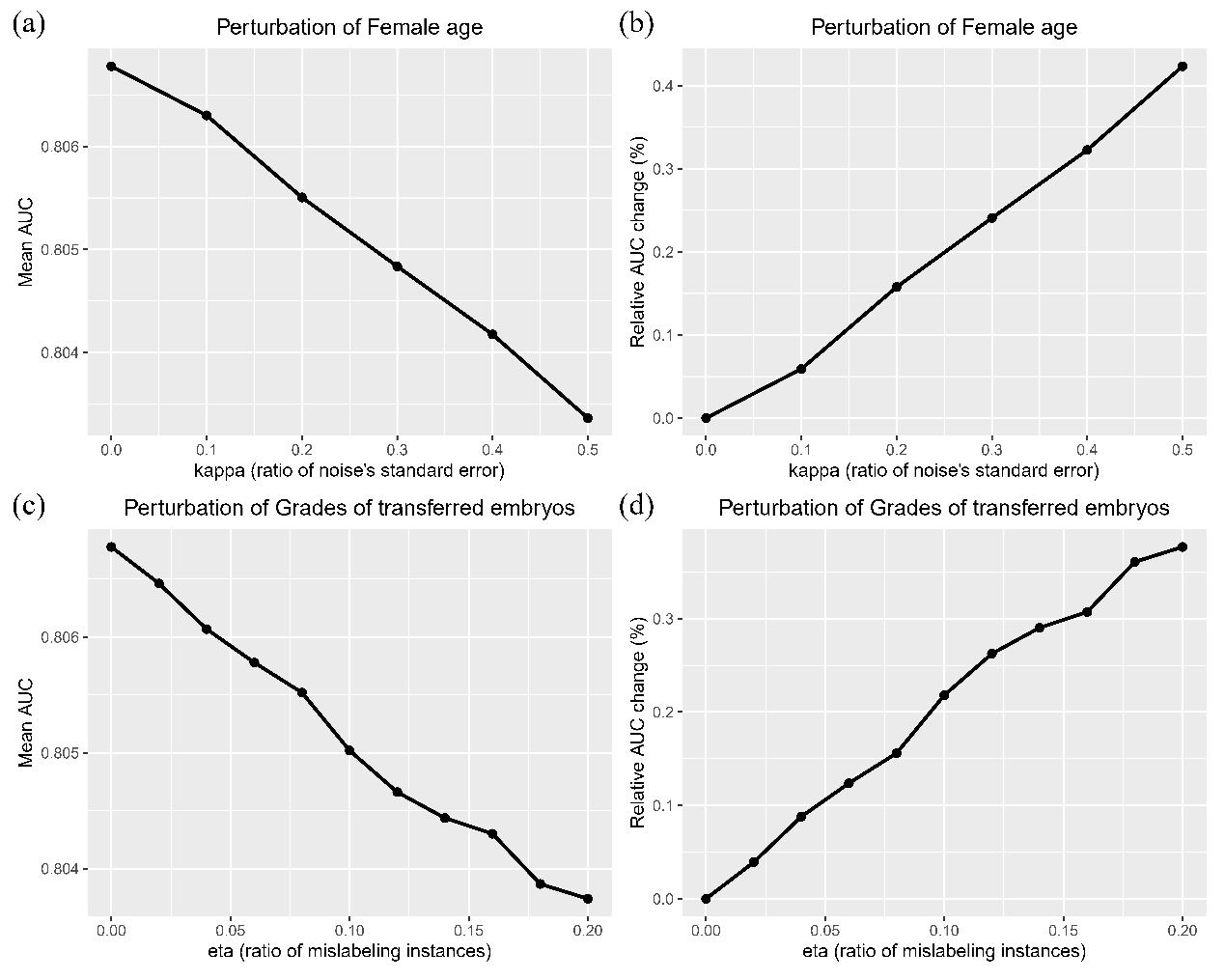


**Fig. S3** Sensitivity analysis of random forest model (trained with 55-feature input and 80% training ratios) under feature perturbations​. (a-b) Mean AUC and relative AUC change (%) after introducing Gaussian noise to female age. (c-d) Mean AUC and relative AUC change (%) following Grades of transferred embryos mislabeling.

**REFERENCES**

Friedman, Jerome H. 2001. Greedy Function Approximation: A Gradient Boosting Machine. Annals of Statistics 29: 1189–1232.

Apley, Daniel W., and Jingyu Zhu. 2020. Visualizing the effects of predictor variables in black box supervised learning models. Journal of the Royal Statistical Society Series B 82 (4): 1059–86. https://doi.org/10.1111/rssb.12377.
